# Impact of Cold Exposure on the Reproductive Function in Female Rats

**DOI:** 10.1155/2018/3674906

**Published:** 2018-11-22

**Authors:** Tong Xu, Xi Li, Lin Yang, Yongqiang Zhang, Li Zhang, Zhan Guo, Xiaodan Cheng, Xiantong Zheng, Baodong Chen, Zhidong Hou, Danfeng Yang

**Affiliations:** ^1^School of Life Science and Engineering, Lanzhou University of Technology, Lanzhou 730050, China; ^2^Tianjin Institute of Environmental and Operational Medicine, Tianjin 300050, China

## Abstract

Female reproductive system diseases caused by exposure to a cold environment are widely considered as important human health challenges. Although the projection of female reproduction in cold temperature has been studied, a holistic view on the probable effects of cold exposure on the functions of the female reproductive system has not been achieved. Our aim was to evaluate the effects of cold exposure to the functions of the ovary and uterus in female rats. For this purpose, female rats were randomly grouped as follows: (1) the cold group was exposed to -10°C, 4 h per day for 2 weeks, and (2) the normal temperature (23 ± 1°C) group was used as control. Alterations were observed in different parameters, including body weight gain, organ coefficients, estrus cycle, and pathology of the cold-exposed female rats. Similarly, the serum reproductive hormones and mRNA expression were evaluated. Cold exposure induced estrus cycle irregularity and some alterations in the morphology of the ovary. Cold exposure impairs the function of the ovary probably by changing the level of serum LH and increasing LHR expression. Cold exposure induced a significant reduction of uterine epithelium height. Cold exposure causes alterations in the morphology of the uterus probably because of the effect of progesterone, the increase in the PR level, and the decrease in the ER level.

## 1. Introduction

Optimum environmental temperature is essential for humans to maintain good health. Extreme cold and heat are harmful to humans. Accumulating evidences showed that the exposure to cold conditions, such as cold air exposure, immersion in cold water, or contacting with cold surfaces may increase human discomfort, impair physical and mental performance, and even lead to higher rate of hospital admission [1–3]. Cold exposure poses a serious threat to public health and is widely considered as a global challenge for human health. Researchers are particularly concerned about female reproductive system disorders, such as hormonal and ovarian tissue disorders, and how to prevent and treat them under cold conditions [4].

The impairment of different set point variables in the physiological system in cold exposure models has been reported. Previous studies have also reported the significant effects of cold exposure on male cardiovascular system, immune system, nervous system, endocrine system, and reproductive system [5–11]. Dorfman et al. observed that cold exposure produces changes in the follicular development in female Sprague-Dawley rats. They found a decrease in prenatal healthy follicles and appearance of a new population of follicles after 2 weeks of cold exposure [12]. Some scholars also found that cold exposure leads to the formation of follicular cysts alongside increased plasma hormone levels, irregular estrus cyclicity, and decreased ovulation in female rats [13, 14]. The estrus cycle in female rats is often used as a biomarker for in vivo hormonal changes and for the pathophysiological status of the ovary and uterus [15]. The estrus cycle in mature rodents is extremely susceptible to multiple factors such as noise, light, environment temperature, and humidity [16]. A previous study showed that the number of growth follicles and mature follicles in female rats with estrous cycle disorder was significantly decreased [17]. In addition, irregular estrus cycle was associated with ovulation problems [18]. Although projection of female reproduction in cold temperature has been studied, a holistic view about the probable effects of cold exposure on the functions of the female reproductive system has not been achieved. Our aim was to evaluate the effects of cold exposure on the functions of the ovary and uterus and understand about the mechanisms in female rats.

## 2. Materials and Methods

### 2.1. Animal Experiments and Ethical Approval

Female Sprague-Dawley rats (210–230 g) were obtained from the Tianjin Institute of Environmental and Operational Medicine (Tianjin, China). The female rats were provided with standard rodent chow and water ad libitum. They were housed under ambient temperature (23±1°C) and humidity (45%–60%) conditions. The female rats were divided into two groups, and the experiment protocols were carried out as described below.

The rats in control group were housed in standard temperature conditions (n=12), and those in the cold group were exposed to -10°C, 4 h per day (10:00 to 14:00; n=12) for 2 weeks. During cold exposure, all rats were fasted and prohibited to consume water. After 2 weeks, surgical procedure was conducted during the diestrus stage. Blood was taken from the abdominal aorta, after which the animals were immediately euthanized. The blood samples were centrifuged for 15 min at 3000 rpm. The sera were separated and stored until the hormone assay.

All procedures involving the care and use of animals were carried out in strict accordance with the guidelines of the research ethics of the Institute of Environmental and Operational Medicine and were approved by the Ethics Review Board of the Institute of Environmental and Operational Medicine. All animals were treated humanely.

### 2.2. Body Weight and Organ Coefficients

The animals' body weights were measured every two days during the experiment until termination at day 14. The ovaries and uteri of each rat were dissected after euthanasia and weighed (wet basis). The following formula was used for the calculation of organ coefficients:

(1) $$\begin{array}{c} organ\;\;coefficients\;\;\left(\;\%\;\right)=\frac{Organ\;\;weight\;\;\left(g\right)}{Body\;\;weight\;\;\left(g\right)}\times 100\% \end{array}$$

See [19].

### 2.3. Observation of Estrus Cycle

Vaginal lavage was collected through pipetting with normal saline (0.9% NaCl). During the exposure period, a small amount of fluid was introduced into the vagina, and one or two drops of the resulting cell suspension were placed onto a glass slide twice per day (8.00 am – 9:00 am; 19:00 pm – 20:00 pm). Then, the dried vaginal smears were stained with hematoxylin and eosin. The smears were observed and captured under a digital photomicroscope.

### 2.4. Histomorphological Analysis

The ovary and uterus from each rat were fixed in 4% tissue fixative and then embedded in paraffin wax. The sections (5 *μ*m) were stained with hematoxylin and eosin. The slides were captured through a digital photomicroscope. Histomorphology of the uterus was performed to assess height of the luminal epithelium.

### 2.5. Serum Hormonal Analysis

The serum levels of luteinizing hormone (LH), follicle-stimulating hormone (FSH), estradiol (E2), prolactin (Prl), and progesterone (P) were determined through radioimmunoassay using a kit (FuRui runze Biotechnology, Beijing, China) according to the manufacturer's instructions.

### 2.6. RNA Extraction and Reverse Transcription-Quantitative Polymerase Chain Reaction (RT-qPCR)

Total RNA of the ovary and uterus of each rat was isolated using RNA Extraction Kit (Takara) and was reversely transcribed into cDNA using a Prime-Script RT reagent kit (Takara). Quantitative PCR was performed with SYBR Green (Takara). The gene expression levels of estrogen receptor (ER), luteinizing hormone receptor (LHR), progesterone receptor (PR), and prolactin receptor (PrlR) in the ovary and uterus were tested. *β*-Actin was used as the control housekeeping gene. The primers were designed as follows: *β*-actin, F-CCTAAGGCCAACCGTGAAAA and R-CAGAGGCATACAGGGACAACA; ER-*α*, F-GCTTATTGACCAACCTGGCAGAC and R-AGGATCTCCAACCAGGCACAC; LHR, F-TCTTGGAAATGCTACACAGCAAG and R-GGAGGAGGGCAAAATACAGAAA; PR, F-TCGTACAAGCATGTCAGTGGACAG and R-CATGGTAAGGCACAGCGAGTAGAA; and PrlR, F-GGTGGAATCCTGGGACAGATG and R-CCAGATGGAAGTGTACTGCTTGCTA. No difference was found in the *β*-actin expression among the groups. The relative expression of genes was determined using the △△-C_t_ method with normalization to the *β*-actin expression.

### 2.7. Statistical Analysis

All values were presented as mean value ± standard error. SPSS 21.0 statistical software was used to calculate statistical evaluations. An independent* t*-test was performed to establish the statistical significance of differences between the experimental groups. Repeated measures ANOVA followed by a Tukey's post hoc test was performed to compare animal's body weight gain.* P* < 0.05 indicated a statistically significant difference.

## 3. Results

### 3.1. Effect of Cold Exposure on Body Weight and Organ Coefficients in Female Rats

**Figure 1:**
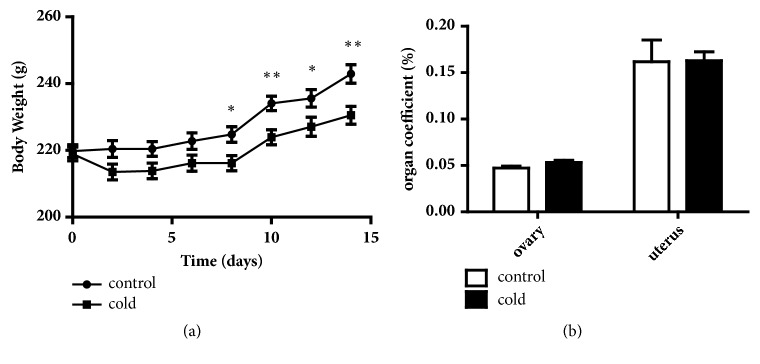
(a) Effects of cold exposure on body weight gain (n=12). (b) Effects of cold exposure on the coefficients of reproductive organs (n=12). Values were presented as the mean value ± standard error. *∗p* < 0.05 and *∗∗p*< 0.01.

The observed body weight gain was relatively normal in the first week (Figure 1(a)). Compared with the control group, the cold exposure group showed significantly reduced rates in body weight gain from day 8 to day 14 (*p* < 0.05 and* p* < 0.01). The coefficients for the ovary and uterus showed no significant differences between the two groups (Figure 1(b)).

### 3.2. Vaginal Smear Cytology and Estrus Cycle

**Figure 2:**
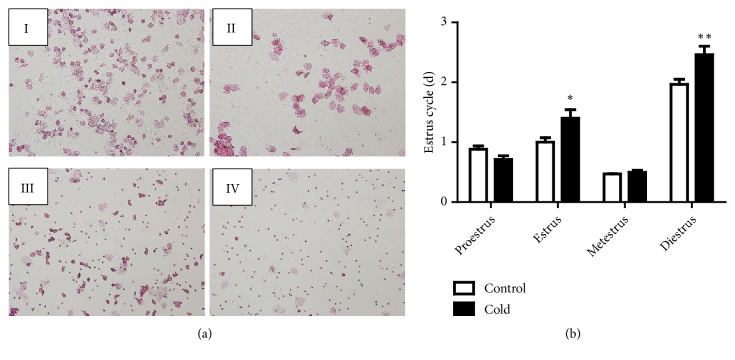
(a) Microphotographs (10×10) of cellular characteristics for the identification of the estrus stage. A proestrus smear mainly consisted of nucleated epithelial cells (I); an estrus smear primarily consisted of anucleated cornified cells (II); a metestrus smear consisted of the same proportion among leukocytes, anucleated cornified cells, and nucleated epithelial cells (III); a diestrus smear primarily consisted of leukocytes (IV). (b) Effects of cold exposure on estrus cycle (n=12). Values were presented as the mean value ± standard error. *∗p* < 0.05 and *∗∗p*< 0.01.

Different stages of estrus cycle (proestrus, estrus, metestrus, and diestrus) were monitored through the observed cellular types from the vaginal smear cytology of female rats (Figure 2(a)). The regular estrus cycle lasted for 4–5 days, including 1 day in proestrus, 1 day in estrus, and 2–3 days in diestrus [20, 21]. Significant changes were observed in each estrus cycle in the cold group compared with the control group (Figure 2(b)). The estrus and diestrus phases were significantly increased after cold exposure (*p* < 0.05 and* p* < 0.01). However, the proestrus and metestrus phases showed no significant difference between the two groups.

### 3.3. Effects of Cold Exposure on the Ovarian and Uterine Histopathology and Uterine Epithelial Height

**Figure 3:**
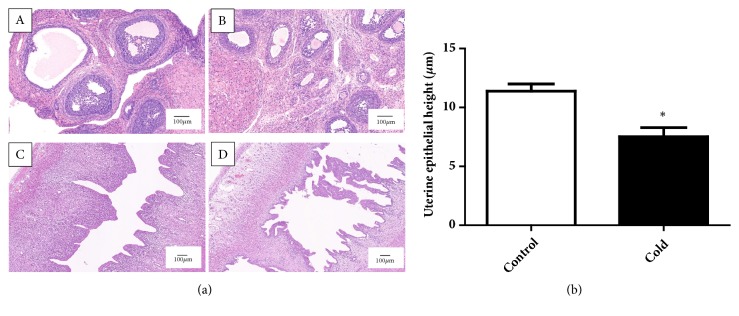
(a) Photomicrographs of rat ovary and uterus by HE staining. Bar = 100 *μ*m. (b) Graphic representations of uterine epithelial height (n=6). Values were presented as the mean value ± standard error. *∗p* < 0.05.

The ovaries of the control group had normal ovarian structure. The follicular and luteal cells in different developmental stages can be seen clearly. The center of the follicle was an oocyte surrounded by granular cells, which were normal and closely arranged. The clear zona and corona radiate were observed between oocytes and granulosa cells (Figure 3A). However, the number of primordial follicles and primary follicles increased significantly in the ovaries of the cold group. We found a significant decrease in the diameter of the ovarian granular layer and theca cell layer in the cold group (Figure 3B).

The analysis of the uterus in the control group showed normal endometrial characteristics (high columnar epithelium and fibrous cell interstitial and gland). The muscular layer and adventitia were histologically characterized by two smooth muscle layers and squamous epithelium on the dense connective tissue (Figure 3C). In the cold exposure group, the lumen of the uterus was narrowed or connected, and the luminal epithelium of the intimal layer was irregular papillary. The endometrium and muscular layer showed edema and decreased or irregular shaped glands (Figure 3D). Histomorphology study demonstrated that the uterine epithelium height in the cold group was significantly lower than that of the control group (*p* < 0.05) (Figure 3(b)).

### 3.4. Effect of Cold Exposure on the Level of Reproductive Hormones

**Figure 4:**
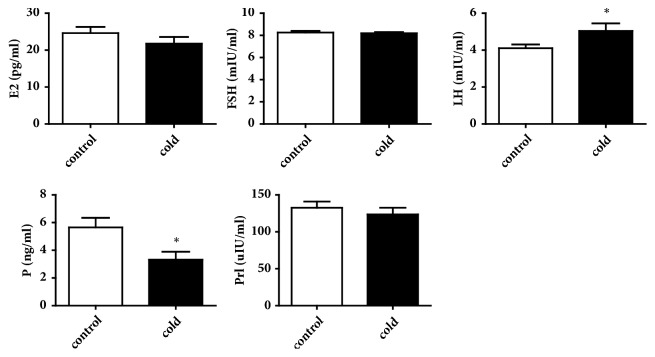
Serum levels of E2, FSH, LH, P, and Prl in control and cold groups of female rats (n=12). Values were presented as the mean value ± standard error. *∗p* < 0.05.

Cold exposure caused a significant reduction serum P levels in female rats (*p* < 0.05). Compared with the control rats, the cold group has significantly higher level of serum LH (*p* < 0.05). Serum E2 and Prl in the cold group had a tendency to decrease. The level of serum FSH showed no significant differences between the two groups (Figure 4).

### 3.5. Gene Expression Levels of Hormone Receptor in Ovary and Uterus

**Figure 5:**
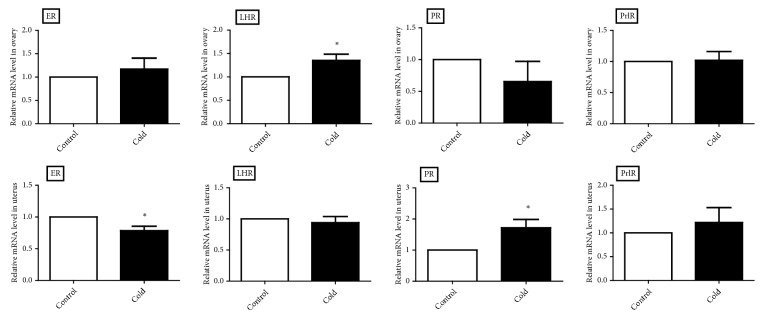
Gene expression of ER, LHR, PR, and PrlR in cold and control groups of female rats (n=6). The level was represented as the mean value ± standard error. *∗p* < 0.05.

We tested the gene expressions of ER, LHR, PR, and PrlR in the ovary and uterus through RT-qPCR. LHR expression was higher (*p* < 0.05) in the ovary in the cold-exposed female rats. A significant increase (*p* < 0.05) in PR gene expression was observed in the uterus of cold-exposed female rats. Cold exposure caused a significant reduction (*p* < 0.05) in the ER gene in the uterus (Figure 5).

## 4. Discussion

Mammalian reproduction is the result of comprehensive regulation of various factors in vivo. Cold temperature is an important environmental factor [22, 23] that leads to changes in reproductive function. These changes represent how animals and humans respond internal and external environment stimuli. Our study was designed to examine the effects of cold exposure on the ovarian and uterine development in female rats. Alterations were observed in different parameters, including body weight gain, organ coefficients, estrus cycle, and pathological change in cold-exposed female rats. Similarly, changes in the levels of serum reproductive hormones and imbalance in mRNA expression were also observed.

Cold exposure acts on energy metabolism in rats [24]. Our previous study showed that cold exposure reduced body weight gain during 4 weeks of treatment in male rats [25]. Cold temperature increases energy expenditure. In present study, the decrease in body weight gain of the cold-exposed female rats coincides with the previous study. In addition, corticotropin-releasing hormone (CRH) release can inhibit food consumption in fasted rats. The stress is one of the reasons why body weight gain decrease in the cold group. It can also affect body weight gain in control rats, and in that event the difference of body weight gain between the control and cold groups would become more remarkable without fast in control rats. Organ coefficient is an indicator reflecting the effect of cold temperature on target organs. However, no significant changes were observed in the organ coefficient of the ovary and uterus.

During the reproductive period, the normal estrus cycle of female rats usually takes 4 – 5 days and contains the proestrus, estrus, metestrus, and diestrus stages. These characteristic stages were distinguished and identified using the cellular types identified through the vaginal smear cytology [21, 26]. The estrus cycle in female rats is often used as a biomarker for in vivo hormonal changes and for the pathophysiological status of the ovary and uterus [15]. Our research on the estrus cycle in cold-exposed female rats showed irregularity. The estrus and diestrus phases were significantly increased after cold exposure. The irregularity in the estrus phases was directly associated with abnormal follicular development in female mice [27]. Furthermore, the estrus cycle might be influenced by the augmented secretion of LH and FSH from the anterior pituitary and the estrogen from the proliferating ovarian follicles in the ovary [15]. Thus, we further investigated the serum levels of the reproductive hormones and other parameters.

The hematoxylin and eosin staining techniques were used to examine the ovarian and uterine histopathology in cold-exposed female rats. Compared with control group, the cold group showed significant pathological change in the ovary and uterus. Cold temperature was known to cause ovarian abnormalities [12, 14]. To our knowledge, this is the first study to report that cold exposure can cause some alterations in the morphology of the uterus in female rats. The uterine environment is important to the successful embryo implantation in mammalian reproduction [26]. In previous reports, reproductive behavior was obviously reduced and resulted in fewer, smaller offspring in extreme circumstances [28, 29]. Therefore, we speculate that cold exposure may lead to a decrease in reproductive capacity due to the impairment of the ovary and uterus.

The reproductive functions of the ovary and uterus can be studied in normal and pathological states under the influences of hormones which were studied. E2 contributes to the development of the follicles and promotes uterine contraction. FSH secreted from the anterior pituitary helps promote maturity of more follicles and physiologically prepares the endometrial formation of the uterus. LH secreted from the anterior pituitary contributes to the development of the corpus luteum and stimulates ovulation. Progesterone secreted by the ovary protects the endometrium. Prl affecting inoculation function is not only secreted in the pituitary, but also secreted within the uterus and other organs [30]. These hormones are crucial to the regulation of the ovarian and uterine environment. The phenomenon that the rising levels of FSH stimulated rising levels of both estrogen and progesterone in proestrus has been observed. The increased estrogen level triggered an LH surge in estrus. Progesterone exhibited a secondary surge in metestrus. Diestrus was characterized by rising estrogen levels [16]. The secretion of Prl occurred during proestrus [31]. We conducted hormone evaluation during the diestrus stage because of its stability and duration. In our research, cold exposure showed increase in LH and decrease in progesterone. Previous study indicated that pesticide exposure decreased the levels of progesterone in female mice and consequently disturbed the uterine receptivity [32, 33]. Similar to the findings of the present study, the progesterone in cold-exposed female rats significantly decreased. Thus, we speculated that the alterations in the levels of serums progesterone may be related to the damage in the uterus, and the change in the levels of serums LH may be associated with the irregularity of the estrus cycle and ovarian abnormalities. However, the hormones are interrelated and interact with each other, and mammalian hormones are also controlled by the hypothalamus and the pituitary gland, presenting the complexity of the female reproductive system [34, 35].

Quantification of gene levels can be used to accurately understand the gene function and alterations in physiological regulation. Quantitative real-time PCR has also been widely used in biological research to assess mRNA expression level because of its advantages, such as accuracy and sensitivity [36, 37]. Activity of reproductive organs depends on the stimulation of hormone through binding to their specific receptors in the ovary and uterus. The ability of estradiol to directly inhibit the secretion of FSH and LH via changes in mRNA levels and stimulate the increase in LHR mRNA expression has been reported [38, 39]. Previous studies indicated that the mRNA level of hormone receptors showed distinct differences with respect to external conditions [37, 40]. Thus, we measured the effect of cold exposure on the hormone receptors in the ovary and uterus. Result showed that the reproductive hormone receptor expression was disrupted at the molecular level in cold-exposed female rats. Uterine epithelial height was established as a highly sensitive marker of estrogenicity in rats [41, 42]. The changes in the uterine epithelial height may be due to the estrogenic properties involving ER even in the absence of estrogen [43] and may also be related to the alteration of PR expression [44]. Increase in the PR level and decrease in the ER level may explain the reduction of uterine epithelial height in our study. LH plays an essential role in reproductive processes. The gonadotropin produces its effects through binding to LHR [45]. Our results were in contrast to those of Patel at el., who observed the increased levels of LH and LHR in POS rats [46]. In our study, cold exposure caused irregular estrus cycle and ovarian abnormalities. These changes were attributed to the high levels of LH and LHR. In addition, the underlying feedback mechanisms in the HPO-axis remain unclear and require further investigation.

## 5. Conclusion

Cold exposure induced estrus cycle irregularity and some alterations in the morphology of the ovary. Cold exposure impairs the function of the ovary probably by changing the level of serum LH and increasing LHR expression. Cold exposure induced a significant reduction of uterine epithelium height. Cold exposure causes alterations in the morphology of the uterus probably because of the effect of progesterone, the increase in the PR level, and the decrease in the ER level.

## Data Availability

The data used to support the findings of this study are available from the corresponding author upon request.
